# Influence of vermicompost and sheep manure on mechanical properties of tomato fruit

**DOI:** 10.1002/fsn3.877

**Published:** 2019-02-27

**Authors:** Ahmad Jahanbakhshi, Kamran Kheiralipour

**Affiliations:** ^1^ Department of Biosystems Engineering University of Mohaghegh Ardabili Ardabil Iran; ^2^ Department of Biosystems Engineering Ilam University Ilam Iran

**Keywords:** pressure test, quality, shear test, sheep manure, tomato, vermicompost

## Abstract

Mechanical properties of the horticultural products play an important role in improving the products quality and storage life after harvesting and also reducing product waste. Recently, using organic fertilizers has increasing trend for producing high‐quality products as well as improvement of soil quality. Two of the best options to produce organic material and sustainability of agricultural production are vermicompost and sheep manure. The present study relied on determination of mechanical properties through pressure and shear tests. Vermicompost and sheep manure were used separately to fertilize the soil. After planting tomato seeds and harvesting, tomato fruits were analyzed by a universal test machine. The results showed that vermicompost was a better fertilizer than sheep manure due to its more appropriate carbon to nitrogen ratio (C/N), acidity, and salinity. Also, in the pressure test, the maximum force required for bruise of tomato produced with vermicompost (41.5N) was more than that of control sample (no fertilizer) and sheep manure. In the shearing test, the maximum force required for shearing tomato produced with vermicompost (58.60 N) was lower than that of control sample (no fertilizer) and sheep manure. The findings of this study can be used to reduce the amount of waste at different stages of tomato production and supply including the design and optimization of processing and transportation equipment.

## 1 INTRODUCTION

In Iran, agricultural products are wasted in range of 5% to 50% at different stages of production to supply (Mirza Kazemi & Gholamalizadeh, 2015; Salehi, Taghizadeh‐Alisaraei, Shahidi, & Jahanbakhshi, 2018). One way to reduce such wastes is improvement of the mechanical properties of agricultural products.

Mechanical properties of agricultural products have an effective role in determining the quality of the products, reducing the potential damage caused in transportation, and eventually designing the equipment used in processing stage of the products (Desmet, Lammertyn, Verlinden, Darius, & Nicolaï, 2004; Ince, Çevik, & Vursavuş, 2016; Jahanbakhshi, 2018; Sitkei, 1987; Zhang et al., 2016).

Prasad and Gupta (1975) studied the mechanical properties of corn stalk. They determined the shearing force and strength of the stalks considering different loading rates. Ince, Uğurluay, Güzel, and Özcan (2005) carried out a study on the flexural and shear properties of sunflower. They found that increasing of moisture content decreases shearing modulus of elasticity and bending stress whereas increases the shearing energy. Krishna and Reddy (2006) investigated on mechanical properties of orange fruit and peel through determining compressive and shearing tests. The results of their study showed that the shearing force and energy of the fruits were decreased by increasing of storage time. Also, mechanical properties of different fruits and vegetables have been investigated such as apple (Kheiralipour et al., 2008), wild pistachio (Heidarbeigi, Ahmadi, Kheiralipour, & Tabatabaeefar, 2008), khinjuk (Heidarbeigi, Ahmadi, Kheiralipour, & Tabatabaeefar, 2009), peppermint stem (Kheiralipour & Hoseinpour, 2016), and carrot (Jahanbakhshi, Abbaspour‐Gilandeh, & Gundoshmian, 2018).

Vermicompost as a type of biological fertilizer is produced through a continuous and slow passage of decaying organic materials in the gastrointestinal tract of some earthworm species. When passing through the earthworm body, the materials are impregnated with the gastrointestinal mucosa, vitamins and enzymes before defecation. Thus, it is used as an enriched and very useful organic fertilizer that improves the quality and fertility of the soil (Haribhushan et al., 2013; Smith, 1998). Rochana, Sawaneg, Patma, and Bunyong (2006) stated that vermicompost leads to improve the size of the soil pores and increase soil porosity and thus the water holding capacity of the soil.

Kashem, Sarker, Hossain, and Islam (2015) investigated the effects of vermicompost and mineral fertilizers on the vegetative growth and production of tomato fruit. They reported that the relationship between vermicompost use during the growth period and plant height, leaf number, shoot and root dry weights, and number of fruits was highly significant at 5% probability level. Feibert, Shock, Barnum, and Saunders (1995) observed that using 15 tons of compost per hectare could increase onion crop yield by 15%. The researchers believed that the enhanced crop yield was due to nutrition improvement and increasing permeability and ventilation as well as the microbial activity in plant root zone. Cook, Keeling, and Bloxham (1997) reported that using vermicompost in a spring barely field caused to increase the plant's dry matter by 25% and it also increased the number of buds per plant. In organic cultivation, El Gendy, Hosni, Omer, and Reham (2001) stated that vermicompost improved the qualitative and quantitative performance of basil plant. It was reported that using vermicompost not only enhances the growth and germination of plants (Atiyeh et al., 2000; Joshi & Vig, 2010; Truong & Wang, 2015) but also enhances the growth of seedling vegetables (Bachman & Metzeger, 1998).

Tomato is the edible fruit of the *Solanum lycopersicum* which belongs to *Solanaceae* family. It is one of the most common commercial vegetables in the world. Due to low levels of fat, calorie, and free cholesterol and high levels of A, B, and C vitamins and carotene and lycopene, tomato fruit and its products are considered as healthy foods in human beings’ daily diets (Alam, Rahman, Mamun, Ahmad, & Islam, 2006; Ghaffari, RezaGhassemzadeh, Sadeghi, & Alijani, 2015; Heuvelink, 2005). Tomato is one of the most important horticultural products in different parts of Iran. Compared to other crops, tomato fruit has high waste due to high moisture content up to 90% (Gould, 2013; Heuvelink, 2005). Knowing the mechanical properties of tomato is useful to predict the conditions result in mechanical damage, and it is very important in optimization of processing machinery. In this regard, researchers have shown that there is a linear relationship between tomato's mechanical properties and its vulnerability (*R* ^2^ = 0.78) (Desmet et al., 2004). Proper nutrition of the horticultural products plays an important role in reducing waste and improving the quality of the products and their storage life after harvesting (Kays, 1991; Wills, McGlasson, Graham, & Joyce, 2007).

So according to above and also due to the importance of theoretical knowledge about the reducing agricultural waste and the lack of reporting on tomato waste, the authors decided to investigate the effect of vermicompost and sheep manure on the mechanical properties of tomato.

## 2 MATERIALS AND METHODS

**Figure 1:**
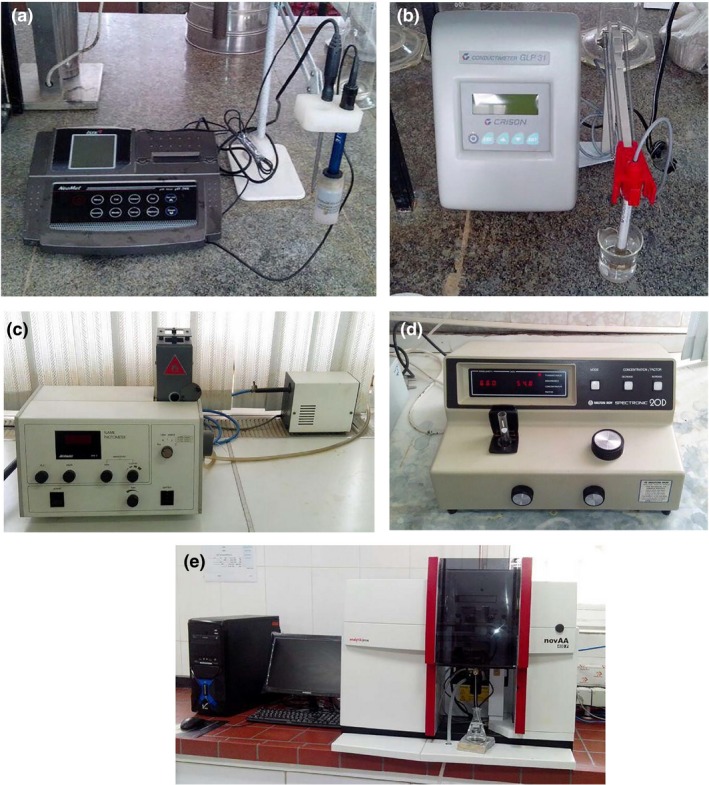
The used instruments to determine chemical properties of the manures. (a) pH‐meter, (b) EC‐meter, (c) Flame photometer, (d) Spectrophotometer, and (e) Atomic absorption spectrometer

To investigate the effects of vermicompost and sheep manure on the mechanical properties of tomato fruit, three treatments including control sample (no fertilizer), vermicompost, and manure fertilizer were considered to be evaluated. Some chemical properties of the vermicompost and sheep manure were determined. The pH of the samples was measured using the PH‐200L digital pH meter (Figure 1a). Crison GLP31 EC meter was applied (Figure 1b) to determine C. K and P of the samples were recorded by Jenway PFP7 flame photometer (Figure 1c) and Spectronic 2OD spectrophotometer (Figure 1d), respectively. Some micronutrients such as Fe, Zn, Cu, and Mn were determined using the Analytikjena nova 400P atomic absorption spectrometer (Figure 1e).

**Figure 2:**
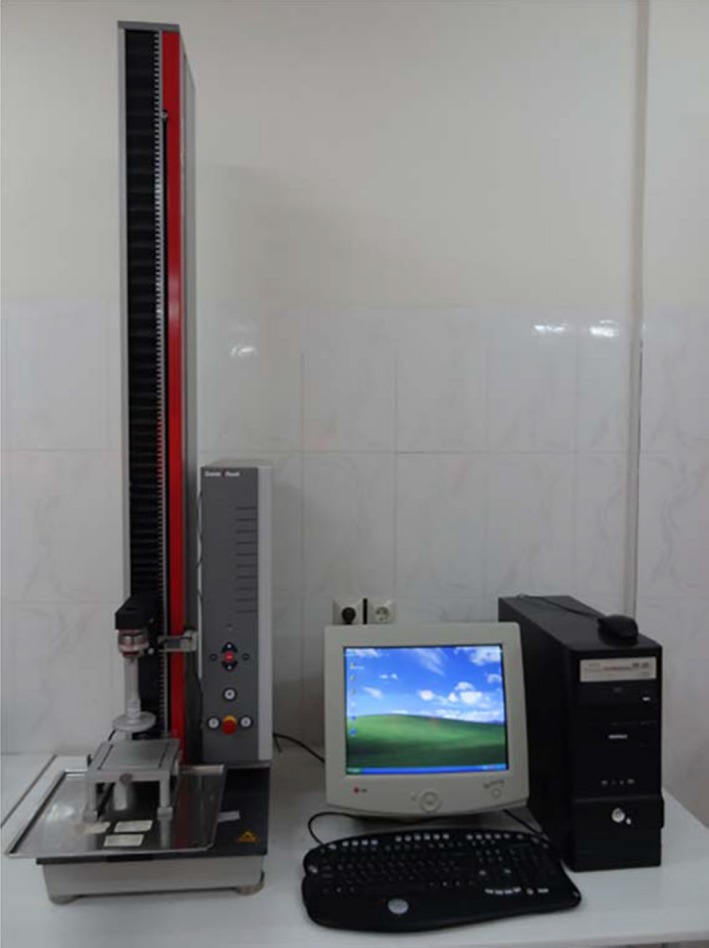
The Zwick/Roell universal testing machine

Each treatment was experimented with three replications. For that, 6 plots with area of 2 m^2^ were prepared. Fertilizers were mixed with soil at the depth of 15 cm with amount of 1 kg in each plot with area of 2 m^2^ (5 tons per hectare). All variables such as irrigation, temperature, fighting against pests and diseases, weed control, and light were the same for all treatments during the growing period. In each plot, three tomatoes were planted at the distance of 30 cm from each other. The ripped tomatoes were hand‐picked, cleaned manually, and stored in an environment with the temperature of 11–12°C. The tomatoes were transferred from the storage place to the laboratory about one hour before determining mechanical properties. To determine the mechanical properties of tomato fruit, two tests, that is, pressure (compress) and shear test, were performed. To do so, a universal testing machine (Model: Z 0.5; Zwick/Roell company, Germany) was used (Figure 2). The mechanical properties were tested on three samples of tomato for each plot.

**Figure 3:**
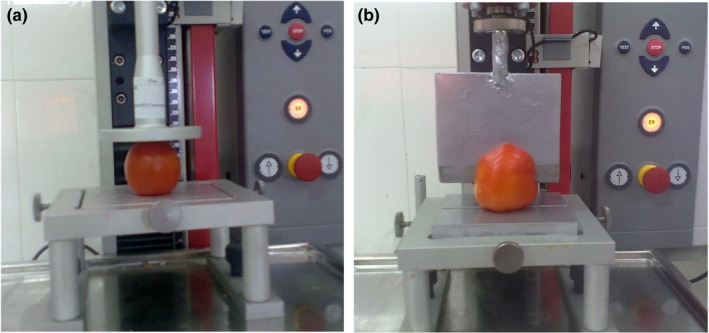
(a) Pressure test and (b) Shear test

Pressure test was conducted under vertical loading according to the ASTMD 790‐03 standard in room temperature at the loading rate of 20 mm/min. At the same loading rate, the shear test was done using a flat edge blade with thickness of 1.4 mm and the blade angle of 30° was used according to DIN53294 standard (Figure 3). The universal testing machine was simultaneously connected to a computer to collect data.

In this research, SAS 9.4 software was used to analyze and perform statistical operations based on completely randomized design. Also, the mean comparison between treatments was done based on LSD test.

## 3 RESULTS AND DISCUSSION

The results of chemical analysis of the vermicompost and sheep manure have been given in Table 1. The results indicated that carbon to nitrogen ratio is more appropriate in vermicompost compared to sheep manure. Thus, using vermicompost as a fertilizer would provide better and more facilitative conditions for the plant to absorb nutritious elements.

**Table 1:** Chemical properties of vermicompost and sheep manure

Property	Unit	Vermicompost	Sheep manure
pH	—	7.3	7.8
EC	dS/m	7.5	11.5
Fe	mg/kg	7,653	7,432
Zn	mg/kg	278	176
Cu	mg/kg	31	25
Mn	mg/kg	475	420
K	%	0.75	0.62
P	%	0.49	0.41
O.C	%	28	19
C/N	—	12.8	28

The ability to absorb nutrients is highly dependent on the pH, and the proper pH range for the crops is between 6.5 and 7.5. According to the results of this study, vermicompost has more neutral pH compared to sheep manure and using a high amount of that has not detrimental effects on the soil and plants.

Moreover, vermicompost is odorless and its impurity is much lower than sheep manure. The EC of the vermicompost is modified much more than the sheep manure and is at such a desirable level that it does not contaminate the soil. In addition, it decreases the salinity stress so causes to avoid change of the amount and types of the regulating metabolites of plant growth and thus decrease its effects on the growth rate.

Another major advantage of vermicompost compared to sheep manure is the high absorbable concentrations of K and P elements. Also, organic acids in vermicompost are mostly organic chelating agents that absorb micronutrients such as Fe, Zn, Cu, and Mn (Ahmad Abadi, Ghajar Sepanlou, & Bahmanyar, 2011; Alikhani, Yakhchali, & Mohammadi, 2011; Dominguez & Edwards, 2010; Hashemimajd, Kalbasi, Golchin, & Shariatmadari, 2004; Tognetti, Laos, Mazzarino, & Hernandez, 2005). Vermicompost and sheep manure are very similar with respect to their advantages but the disadvantages of sheep manure are more than those of the vermicompost. Some of these disadvantages are the existence of different types of viruses and microorganisms pathogens, higher crop production cast due to hiring workers and use pesticides to control the weeds in gardens and farms, more undesirable microorganisms, difficult storage process, creation of disgusting smell, and the transmission of common diseases between humans and animals.

The results of variance analysis of the effect of fertilizer type on the mechanical properties of tomato in the pressure test have been presented in Table 2. The results show that the effect of fertilizer type on mechanical properties of tomatoes was significant in the pressure test at 1% probability level. This result indicates that it can be said that there is a significant difference between the mean treatments with 99% confidence.

**Table 2:** Results of variance analysis of the effect of fertilizer type on mechanical properties of tomato in the pressure test

Factor	DF			Mean square		
	Peak force	Deformation	Energy	Peak force	Deformation	Energy
Fertilizer	2	2	2	93.17**	121.76**	246,850.03**
Error	6	6	6	0.48	0.12	48.13
Total	8	8	8	—	—	—

^**^Significant at 1% probability level.

The mechanical properties of tomato determined through pressure test have been listed in Table 3. In this table, the values of the peak force required to the failure of the tomato fruit, deformation in peak force, and the energy to reach the peak force under pressure test have been provided.

**Table 3:** Mechanical properties of tomato in pressure test

Pressure property	Manure	Minimum	Average	Maximum	Standard deviation	Coefficient of variation (%)
Peak force (N)	Control sample	30.20	30.60^c^	31.00	0.40	1.30
	Sheep manure	33.60	34.03^b^	34.50	0.45	1.32
	Vermicompost	40.80	41.50^a^	42.70	1.04	2.50
Deformation (mm)	Control sample	13.15	13.45^b^	14.00	0.47	3.49
	Sheep manure	13.83	14.11^b^	14.50	0.34	2.40
	Vermicompost	24.60	24.80^a^	25.00	0.20	0.80
Energy (N mm)	Control sample	203.35	207.65c	210.11	3.73	1.79
	Sheep manure	219.00	223.31^b^	226.21	3.80	1.70
	Vermicompost	699.89	712.13^a^	720.12	10.76	1.51

Notes

Nonsimilar letters indicate a significant difrence at 1% probablity level.

The mean values of the peak force, deformation, and the energy to reach the peak force under pressure test were 30.60 N, 13.45 mm, and 207.65 N/mm, respectively, for the fruits produced without fertilizer (control samples), 34.03 N, 14.11 mm, and 223.31 N/mm, respectively, for the fruits produced by sheep manure and 41.50 N, 24.80 mm, and 712.13 N/mm, respectively, for the fruits produced by vermicompost fertilizer. The results showed that the peak force of tomato produced using vermicompost was 45.05% and 42.44% more than that of control sample (no fertilizer) and sheep manure, respectively. Also, the deformation was more in vermicompost. So, the energy performed to reach the peak force of the tomato fruit produced by vermicompost was higher than that of control sample (no fertilizer) and sheep manure. This is due to rich nutrients in vermicompost that gradually provides the plant requirements, and this makes the soil more fertile and yields higher‐quality products. Therefore, it is suggested that farmers use organic fertilizers such as vermicompost in order to reduce tomato waste at different stages of harvesting and supply chain.

In similar studies, Truong and Wang (2015), Kashem et al. (2015), Tognetti et al. (2005), Alikhani et al. (2011) and Ahmad Abadi and Ghajar Sepanlou (2012) confirmed that besides better physical and chemical properties of vermicompost compared to ordinary composts, it is particularly superior to provide plant growth stimulants such as vitamins, enzymes, and growth factors.

The results of variance analysis of the effect of fertilizer type on the mechanical properties of tomato in the shear test have been presented in Table 4. The results showed that the effect of fertilizer type at the 1% probability level on the shear test of tomatoes was significant.

**Table 4:** Results of variance analysis of the effect of fertilizer type on mechanical properties of tomato in the shear test

Factor	DF			Mean square		
	Maximum force	Deformation	Shear resistance	Maximum force	Deformation	Shear resistance
Fertilizer	2	2	2	1,102.02**	1,759.19**	0.0003**
Error	6	6	6	0.83	2.72	0.000001
Total	8	8	8	—	—	—

^**^Significant at 1% probability level.

The shearing properties of tomato fruit have been given in Table 5. In the shear test, the maximum force required to cut the tomato fruit, shear resistance, and deformation while exercising the maximum force of tomatoes produced without fertilizer were 93.26 N, 62.70 mm, and 0.043 N/mm^2^, respectively, 90.10 N, 59.63 mm, and 0.036 N/mm^2^, respectively, for the tomatoes produced by sheep manure and 58.60 N, 19.31 mm, and 0.023 N/mm^2^, respectively, for the tomatoes produced by vermicompost fertilizer. As can be seen, the maximum force necessary to shear the tomato produced by vermicompost is lower than that of control sample (no fertilizer) and sheep manure. The data obtained about the mechanical properties of tomato in the shear test can be used by the factories that process this product. These results are in agreement with the results obtained by Jahanbakhshi et al. (2018), Sessiz, Esgici, Özdemir, Eliçin, and Pekitkan (2015), Jafari, Rajabipour, and Mobli (2010), and Persson (1987).

**Table 5:** Mechanical properties of tomato in shear test

Shear property	Manure	Maximum	Average	Minimum	Standard deviation	Coefficient of variation (%)
Maximum force (N)	Control sample	94.45	93.26^a^	92.20	1.12	1.20
	Sheep manure	91.20	90.10^b^	89.10	1.05	1.16
	Vermicompost	59.00	58.60^c^	58.35	0.35	0.59
Deformation (mm)	Control sample	64.95	62.70^a^	61.50	1.95	3.11
	Sheep manure	61.85	59.63^a^	58.10	1.96	3.28
	Vermicompost	20.13	19.31^b^	18.8	0.71	3.67
Shear resistance (N/mm^2^)	Control sample	0.045	0.043^a^	0.042	0.001	2.32
	Sheep manure	0.037	0.036^b^	0.035	0.001	2.77
	Vermicompost	0.024	0.023^c^	0.022	0.0003	1.30

Notes

Nonsimilar letters indicate a significant difrence at 1% probablity level.

## 4 CONCLUSIONS

In the present study, pressure and shear properties of tomato fruit produced using control sample (no fertilizer), vermicompost, and sheep manure were determined. The pressure properties of the tomato fruits produced by vermicompost were higher than that of control sample (no fertilizer) and sheep manure whereas the shearing properties of the fruits produced by vermicompost were lower than that of control sample (no fertilizer) and sheep manure. It was concluded that vermicompost can be used to improve the mechanical properties and then reduce the amount of waste at different stages of tomato production and supply including.

## CONFLICT OF INTEREST

The authors have declared no conflict of interest.

## ETHICAL REVIEW

This study does not involve any human or animal testing.
